# Supplementary material for: Phytopathological Threats Associated with Quinoa (Chenopodium quinoa Willd.) Cultivation and Seed Production in an Area of Central Italy
Source: Plants (Basel). 2021 Sep 16;10(9):1933. doi: 10.3390/plants10091933 (PMC8467509; doi:10.3390/plants10091933)
Supplement: Supplementary file 1 [file plants-10-01933-s001.zip › Supplementary Table S1.pdf]

Supplementary Table S1. Isolates of *Fusarium* species belonging to the *Fusarium incarnatum-equiseti* species complex (FIESC) used in the phylogenetic analysis and related GenBank accession numbers and clade.

Phylogenetic species	Isolates	*tef1α* GenBank accession numbers	Clade
FIESC 1-a	NRRL 43640	GQ505667	*Equiseti*
FIESC 2-a	NRRL 36401	GQ505651	*Equiseti*
FIESC 3-a	NRRL 36318	GQ505646	*Equiseti*
FIESC 4-a	NRRL 20423	GQ505593	*Equiseti*
FIESC 5-a	NRRL 32871	GQ505619	*Equiseti*
FIESC 5-b	NRRL 45995	GQ505670	*Equiseti*
FIESC 5-c	NRRL 25795	GQ505597	*Equiseti*
FIESC 5-d	NRRL 34035	GQ505637	*Equiseti*
FIESC 5-f	NRRL 45997	GQ505672	*Equiseti*
FIESC 6-a	NRRL 43638	GQ505665.	*Equiseti*
FIESC 7-a	NRRL 32997	GQ505624	*Equiseti*
FIESC 8-b	NRRL 43498	GQ505658	*Equiseti*
FIESC 9-c	NRRL 26922	GQ505601	*Equiseti*
FIESC 10-a	NRRL 3020	GQ505586	*Equiseti*
FIESC 11-a	NRRL 36372	GQ505649	*Equiseti*
FIESC 12-c	NRRL 36393	GQ505645	*Equiseti*
FIESC 13-a	NRRL 43635	GQ505662	*Equiseti*
FIESC 14-a	NRRL 26419	GQ505599	*Equiseti*
FIESC 15-b	NRRL 32182	GQ505611	*Incarnatum*
FIESC 16-c	NRRL 43730	GQ505669	*Incarnatum*
FIESC 17-a	NRRL 32864	GQ505613	*Incarnatum*
FIESC 18-a	NRRL 31167	GQ505608	*Incarnatum*
FIESC 19-a	NRRL 43639	GQ505666.	*Incarnatum*
FIESC 20-a	NRRL 34003	GQ505627	*Incarnatum*
FIESC 21-a	NRRL 13335	GQ505590	*Incarnatum*
FIESC 22-a	NRRL 34002	GQ505626	*Incarnatum*
FIESC 23-a	NRRL 32867	GQ505616	*Incarnatum*
FIESC 24-b	NRRL 43297	GQ505657	*Incarnatum*
FIESC 25-c	NRRL 32868	GQ505617	*Incarnatum*
FIESC 26-b	NRRL 28714	GQ505598	*Incarnatum*
FIESC 27-a	NRRL 20722	GQ505595	*Incarnatum*
FIESC 28-a	NRRL 28577	GQ505603	*Incarnatum*
FIESC 29-a	NRRL 25084	JF740715	*Equiseti*
FIESC 30-a	NRRL 52758	JF740833	*Equiseti*
**FIESC-PG-Q1**	-	**MZ191105**	*Equiseti*
Fusarium concolor	NRRL 13459	GQ505674	-

NRRL: Mycological collection of the National Regional Research Laboratory, Peoria, IL, USA. Bold type, isolate and accession number obtained in the present study.
