# Supplementary material for: Phytopathological Threats Associated with Quinoa (Chenopodium quinoa Willd.) Cultivation and Seed Production in an Area of Central Italy
Source: Plants (Basel). 2021 Sep 16;10(9):1933. doi: 10.3390/plants10091933 (PMC8467509; doi:10.3390/plants10091933)
Supplement: Supplementary file 1 [file plants-10-01933-s001.zip › Supplementary Table S2.pdf]

Supplementary Table S2. Isolates of *Peronospora* species used in the phylogenetic analysis and related host, origin and GenBank accession numbers.

Species	Isolates[1]	Host	Origin	*ITS* GenBank Accession Numbers
Peronospora boni-henrici	WU 22886	*Chenopodium bonus-hericus*	Austria	AY198286
	SOMF 15654	*Chenopodium bonus-hericus*	Bulgaria	EF614952
	BPI 787221	*Chenopodium bonus-hericus*	Germany	EF614953
	BPI 787219	*Chenopodium bonus-hericus*	Switzerland	EF614954
Peronospora chenopodii	2001 HAL	*Chenopodium hybridum*	Germany	EF614955
Peronospora chenopodii-polyspermi	WU 22891	*Chenopodium polyspermum*	Austria	AY198291
	BPI 787538	*Chenopodium polyspermum*	Germany	EF614956
Peronospora effusa	BPI 877761	*Spinacia oleracea*	Korea	DQ643876
Peronospora farinosa s.l.	RD 1459	*Atriplex* sp.	Argentina	EU571109
	1962 UPS	*Atriplex* sp	Iceland	DQ643840
	BPI 789214	*Atriplex littoralis*	Sweden	DQ643841
	1962 UPS	*Atriplex patula*	Sweden	DQ643842
Peronospora manshurica	KUS-F17669	*Glycine soja*	Korea	AY211019
Peronospora variabilis	RD 90	*Chenopodium album*	Argentina	EU571110
	HMAS 57036	*Chenopodium album*	China	EF614959
	BPI 791617	*Chenopodium album*	Germany	EF614961
	1935 UPS	*Chenopodium album*	Ireland	EF614962
	BPI 791615	*Chenopodium album*	Italy	EF614963
	BPI 791620	*Chenopodium album*	Latvia	EF614967
	2000 UPS	*Chenopodium album*	Romania	AF465762
	1932 UPS	*Chenopodium album*	Netherland	EF614968
	RD 1436	*Chenopodium quinoa*	Argentina	EU571113
	BPI 788269a	*Chenopodium quinoa*	Bolivia	EU571108
	DK03	*Chenopodium quinoa*	Denmark	FJ638473
	DK04	*Chenopodium quinoa*	Denmark	EU113306
	DK05	*Chenopodium quinoa*	Denmark	FJ638474
	DK06	*Chenopodium quinoa*	Denmark	FJ638475
	DK07	*Chenopodium quinoa*	Denmark	FJ638476
	DK08	*Chenopodium quinoa*	Denmark	FJ638477
	DK10	*Chenopodium quinoa*	Denmark	FJ638478
	DK11	*Chenopodium quinoa*	Denmark	FJ638479
	EC04	*Chenopodium quinoa*	Ecuador	EU113303
	EC05	*Chenopodium quinoa*	Ecuador	FJ638491
	EC11	*Chenopodium quinoa*	Ecuador	EU113309
	PE01	*Chenopodium quinoa*	Peru	FJ638496
	PE02	*Chenopodium quinoa*	Peru	EU113304
	PE03	*Chenopodium quinoa*	Peru	FJ638497
	**PV-PG-Q1**	*Chenopodium quinoa*	Italy	**MZ191106**

[1]**BPI**: Systematic Mycology and Microbiology Laboratory, USDA-ARS, Beltsville, Maryland, U.S.A.; **HAL**: Herbarium of Institut für Biologie, Department of Geobotany and Botanical Garden, Martin-Luther-University, Halle, Germany; **HMAS**: Herbarium Mycologium, Institute of Microbiology, Chinese Academy of Sciences, Chaoyang, People's Republic of China; **KUS**: Herbarium of School of Life Sciences, Korea University, Seoul, Republic of Korea; **RD**: R. Delhey (private collections) in the Phytopathology Lab of Bahia Blanca, Argentina; **SOMF**: Mycological Collection, Institute of Biodiversity and Ecosystem Research, Bulgarian Academy of Sciences, Sofia, Bulgaria; **UPS**: Museum of Evolution, Uppsala University, Uppsala, Sweden; **WU**: Herbarium of the Institute of Botany, University of Vienna, Austria. Isolated listed as **DK**, **EC** and **PE** are from Reference [50]. Bold type, isolate and accession number obtained in the present study.
