# Supplementary figures and images for: Phytopathological Threats Associated with Quinoa (Chenopodium quinoa Willd.) Cultivation and Seed Production in an Area of Central Italy
Source: Plants (Basel). 2021 Sep 16;10(9):1933. doi: 10.3390/plants10091933 (PMC8467509; doi:10.3390/plants10091933)

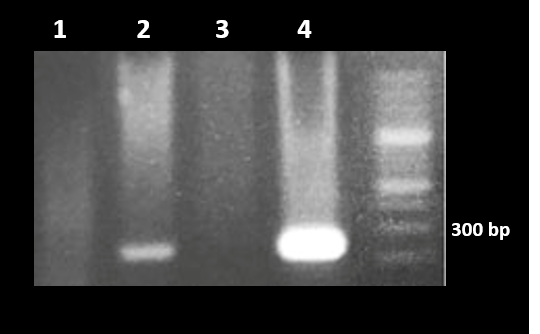

Supplement: Supplementary file 1 [file plants-10-01933-s001.zip › Supplementary Figure S1.tif]
